# Restless Wanderers

**Published:** 1866-11

**Authors:** 


### RESTLESS WANDERERS.

We are moved to pity many times in meeting with a class of men who are seeking for, they know not what. They see evil in the world and sorrow; they see oppression and degradation, and while observing them, feel the more, in that they have experiences in the same directions; tearful, bitter, almost heart-breaking experiences, it may be, and in blindness and powerlessness they are groping about wearily and painfully for a remedy.

In all these, not a single man or woman is found who does not begin by attacking the present system of received religion. Most of them persuade themselves that they believe the Bible, and readily refer to it as confirmatory of their peculiar systems, but in every case, they will only consent that the holy book shall be interpreted according to some preconceived views of their own. They are quite willing to make the Bible their arbiter, the tribunal of last resort, but then they insist that they must have the interpretation of its meaning. Yet with all this, they are dissatisfied and unhappy; there is a feeling of unrest which is devouring them, and they will talk *ad infinitum* to everybody, inferring from admissions of the occasional good sentiments which they avow, a more or less implied assent to their whole system, and drawing some comfort therefrom, they arrive at the conclusion that the whole world is rapidly falling into their views; and soon fanaticism assumes its sway, to hurry them to still greater extremes, until they are dashed on the rocks of suicide, of lunacy, or of perdition.

All these people look sad; they are extremely excitable; they fire up on the instant; and in all, we never fail to see a degree of bitterness towards opponents, and especially is a bitterness exhibited towards ministers, and churches, and communities, in proportion as these appear thriving, prosperous, and happy. Nor is this all; the rich are their universal anvil; on it they pound most mercilessly. With them, the selfishness of the rich is an exhaustless theme; or, if they ever come to a conclusion, it is this, that if these same rich people would commit the distribution of their property to them, the millenium would come in a very few days; and while handling the money which they never had the capacity to earn or keep, they would be the happiest people on the face of the earth, and would thence assume that everybody else was prosperous and happy too; just as a short time before, they had concluded that everybody was poor, and wretched, and miserable, because they were so themselves.

